# Teaching plain language to medical students: improving communication with disadvantaged patients

**DOI:** 10.1186/s12909-021-02842-1

**Published:** 2021-07-28

**Authors:** Doron Sagi, Sivan Spitzer-Shohat, Michal Schuster, Ligat Daudi, Mary Catharine Joy Rudolf

**Affiliations:** 1 Department of Population Health, Azrieli Faculty of Medicine, Bar-Ilan University, 8 Henrietta Szold, 1311502 Safed, Israel; 2 MSR- The Israel Center for Medical Simulation, Ramat-Gan, Israel; 3 Center for Health and the Social Sciences, University of Chicago, Chicago, USA; 4 Faculty of Humanities, University of the Free State, Bloemfontein, South Africa

**Keywords:** Doctor patient communication, Health literacy, Plain language, Written communication, Experience-based learning, Undergraduate medical education

## Abstract

**Background:**

Low health literacy underpins health inequality and leads to poor adherence to medical care and higher risk of adverse events and rehospitalization. Communication in plain language, therefore, is an essential skill for health professionals to acquire. Most medical education communication skill programs focus on verbal communication, while written communication training is scarce.

ETGAR is a student delivered service for vulnerable patients after hospital discharge in which, amongst other duties, students ‘translate’ the medical discharge letters into plain language and share them with patients at a home visit. This study ascertains how this plain language training impacted on students’ written communication skills using a tool designed for purpose.

**Methods:**

Students, in pairs, wrote three plain language discharge letters over the course of a year for patients whom they encountered in hospital. The students handed over and shared the letters with the patients during a post-discharge home visit. Structured feedback from course instructors was given for each letter. An assessment tool was developed to evaluate students’ ability to tell the hospitalization narrative using plain and clear language. First and last letters were blindly evaluated for the entire cohort (74 letters; 87 students).

**Results:**

Students scored higher in all assessment categories in the third letters, with significant improvement in overall score 3.5 ± 0.8 vs 4.1 ± 0.6 Z = -3.43, *p* = 0.001. The assessment tool’s reliability was high α = 0.797, it successfully differentiated between plain language categories, and its score was not affected by letter length or patient’s medical condition.

**Conclusions:**

Plain language discharge letters written for real patients in the context of experience-based learning improved in quality, providing students with skills to work effectively in an environment where poor health literacy is prevalent. ETGAR may serve as a model for learning written communication skills during clinical years, using the assessment tool for formative or summative evaluation.

## Background

Doctor-patient communication is central to medical care and one cannot underestimate its effect on quality of care [1, 2]. Information gathering, explanation and shared decision making are crucial components for good doctor-patient communication [3]. Plain and understandable language is required to meet these tasks and is especially needed when taking care of patients with low health literacy who have trouble understanding their medical condition and treatment [4, 5]. The U.S. Department of Health and Human services define health literacy as “the degree to which individuals have the ability to find, understand, and use information and services to inform health-related decisions and actions for themselves and others.” [6] According to the WHO, health literacy is not just the ability to read pamphlets, but is critical to empowerment [7]. .Studies have found that poor health literacy is associated with a lack of adherence to medication and treatment [4], higher risk for rehospitalizations [8] and poorer patient outcomes [9].

Communication skills programs are to be found in almost all medical schools, underpinned by educational consensus and curriculum frameworks [10, 11], as well as in models for patient-centered communication [12, 13]. However, most medical educational communication programs focus on doctor-patient encounters, emphasizing face to face interaction and the importance of verbal and nonverbal communication. Only a few educational programs teach written communication [14, 15].

Transition from hospital to home is a critical phase in a patient’s journey, where health status and treatment may change as the patient transfers from the monitored hospital environment to self-care and management in the community [16]. At this vulnerable time post discharge adverse events and rehospitalizations are more likely to occur in patients with low health literacy [8] and other social determinants of health [17] leading to widening disparities and health inequity [18].

Doctor-patient verbal and written communication during transition in care are important for safe discharge of patients back home [19]. The information patients receive and remember is critical for their future self-care and health [20, 21]. The discharge letter serves as the main handover tool. Directed at another doctor and using language rich in medical terminology and jargon, it is indecipherable to most lay people. Intervention programs aimed at improving the safety and quality of transition in care address this gap by emphasizing the importance of clear communication [22, 23] and through design of personalized discharge letters [24].

Since 2016, all clinical students at the Azrieli Faculty of Medicine at Bar-Ilan University take part in the ETGAR^1^ course, a student-delivered service for disadvantaged patients during their transition in care [25]. As part of the service, the students write plain language discharge letters, reformulated from the formal medical letter, and give them to the patients during a post discharge home visit [25].

Through writing plain language discharge letters to real patients during their transition in care and sharing them in the home setting, ETGAR aims to firstly provide students with written communication skills training, and also to promote the understanding of health literacy and other social determinants affecting patient’s health and health inequity [14, 26, 27].

Given the lack of information regarding learning culturally appropriate, written communication skills, we resolved to explore whether the ETGAR experience increased students’ competence in plain language writing to a level appropriate for patients with low health literacy. In order to reliably assess their skills, we developed a tool to analyze their plain language discharge letters over the course of one academic year. Our aim was to ascertain whether this long-term experience-based program improved students’ written communication skills.

## Methods

### Setting and participants

The Azrieli Faculty of Medicine is located in the Galilee in Israel’s Northern periphery, and is home to diverse communities of Jews and Arabs, with high levels of poverty and low levels of educational attainment [28]. Many patients have inadequate Hebrew, the primary language spoken in Israel, either because they are Arab or are immigrants [29]. The ETGAR program is successful in involving disadvantaged patients in terms of these parameters [27].

All 96 first clinical year students participated. They worked in pairs and made visits to the homes of three patients over the course of a year, with the purpose of providing the plain language discharge letter and checking on medication and patients’ condition at home. The patients are selected by hospital staff for their psychosocial vulnerability to readmission [25]. One patient was chosen in each long rotation: Pediatrics, Surgery and Obstetrics/Gynecology. The order of the rotations varies, so that some students start in pediatrics, but end with surgery or gynecology and vice versa.

### Training in written communication skills

At the start of the course, students learn and practice plain language writing as part of a day long training session, followed by a tutorial focusing on writing skills in their first rotation, usually before they have written their first letter.

The students are instructed to ‘translate’ the medical discharge letter and it is made clear that they need to go beyond the technicality of simplifying medical terms. They are encouraged to reorganize the information, set priorities and decide what to include in the letters in order to make them understandable and memorable for the patient.

In developing our student guidance, we followed the PLAIN (Plain Language Action and Information Network) guidance [30] which defines plain language as “communication your audience can understand the first time they read or hear it” [30], namely writing that is clear, concise and well-organized manner. We utilized guidance for plain language writing [31, 32] which includes using the second person and active tense, and avoiding abbreviations, long words and unexplained medical jargon. We instructed students to deliver the hospitalization story as a narrative, presenting the more important occurrences first. To organize the letter using paragraphs, each describing only one topic built from short sentences with one idea per sentence. We also advised students to combine patient education when appropriate, usually through providing the rationale for tests and treatments.

**Fig. 1:**
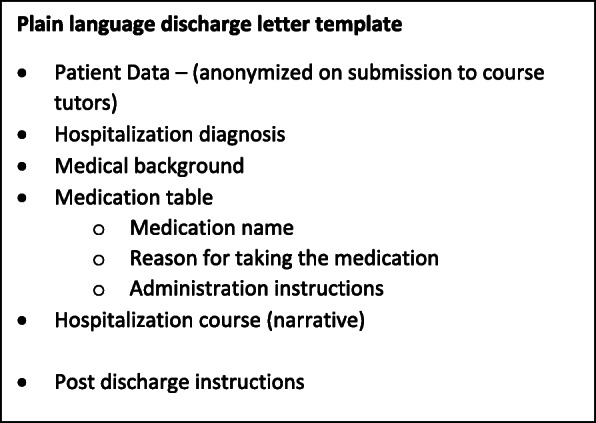
Plain language discharge letter template

We developed a plain language discharge letter template to meet our students and patient needs (Fig. 1), based on templates used for personalized discharge letters [24]. Before conducting the home visit, a clinical tutor on the ward checks the plain language letter for clinical accuracy to avoid medical or pharmacological errors. The tutors did not intervene or amend the students’ letter.

At the end of each rotation, each pair of students submit their plain language discharge letter and a home visit report. They have the opportunity to appreciate and improve the quality of their letters by taking into account the patient’s reaction to the letter at the visit and subsequently learn from the structured feedback they receive on each letter from their course tutor. Each letter is given a mark, which contributes to students’ course grades [25].

### Development of a written plain language assessment tool

In order to consistently and rigorously assess students’ discharge letters we developed an assessment tool based on insights from Keely et al. [33], CanMed teaching and assessment tool guide [32], Đujić et al. [14] and Buurman et al. [24]. It consists of six items: 1) information organization ‘telling the hospitalization narrative’, 2) Adequacy and clarity of medical terms, 3) Language and style, 4) background medical problems, 5) medication table structure and content and 6) post discharge instructions. The scoring is on a 1–5 scale (1 poor, 5 very good). The tool and its rubric are shown in Table 1.

**Table 1:** Plain language discharge letter assessment format and rubric

	Hospital Department
Letter Attributes	Number of sentences Number of Words
Patient’s Data	Number of medications listed in the letter Background diseases (list) Admission diagnoses
*Give a summary score on a likert scale of 1–5*	
**1. Telling the narrative of the hospital admission [1 …….5]**	• Providing the narrative in a logical and helpful order starting with the most important ideas • Describing the main hospitalization phases from admission to discharge • Writing the narrative in an engaging way • Thoughtful inclusion or omitting of medical information to the narrative • Ending with clear guidance as to what the patient needs to do now • Combining patient education, explaining results’ meaning and treatment rationale
**2. Adequacy and clarity of medical terms [1…….5]**	• Good, simple correct explanation of medical and technical terms in a readable manner • Logical description of history, physical examination, and investigations, and why they were done • Avoiding expressions that could be misunderstood by lay people
**3. Language and style [1…….5]**	• Written to the patient (or parent) in the active tense, respectful and not condescending • Written in full sentences using sub-headings when the letter is long • Using short sentences / avoiding long and complex sentences. • No inappropriate phrases (offensive or expressing disrespect) • Inclusion of useful diagram if helpful
**4. Medical problems list [1…….5]**	• A complete list of medical problems, including chronic diseases • The current problem, admission diagnosis - at the top of the list
**5. Medication Table [1…….5]**	• Adherence to the medication table guidelines ○ Nam ○ Concise explanation of action ○ Dosage and administration instructions
**6. Instructions [1…….5]**	• Clear and specific (what to do, when and where) • Providing explanation for referrals and follow up in the community

*Telling the hospitalization narrative* refers to providing the hospital course in a narrative form, emphasizing the main phases of the hospitalization and treatment reflecting the time line of hospitalization [34]. Higher marks are given for starting with the more important ideas, describing the main hospitalization phases and combining patient education into the narrative such as giving the rationale for tests and treatment [32].

The item on *adequacy and clarity of medical terms* looks for avoidance of medical jargon, explanation of medical and technical terms and avoiding terms which could be misunderstood by lay people [14]. The *language and style rubric* looks for writing directly to the patient (in the second person), using the active tense and plain but respectful language. Positive features include writing in short sentences avoiding excessively long sentences or words, using one idea per sentence, and combining the sentences into paragraphs relating to one topic at a time [32, 33].

The *medical background* item seeks the hospitalization diagnosis and a full list of the patient’s medical conditions. The *medication table* rubric looks for a short and clear explanation of the use of each medication, and administration instructions. The *instructions* item looks for a concise clear explanation of referrals and why they are needed and specificity regarding symptoms which are concerning enough to require help. The six items were divided into three categories: telling the narrative- item 1, plain and clear language- items 2,3 and structure- items 4–6.

### Assessing the simplified discharge letters

We identified all letters submitted by students at the end of the first and last rotations in the academic year 2016–2017 to assess change over time. Since the clinical rotation order varied for the cohort, potential bias relating to differing complexity of medical conditions (eg surgical vs medical admissions) was avoided. In order to refine the tool, four investigators (DS, SS, MR and LD) analyzed four random letters to check the rubric and for initial calibration. Discrepancies were discussed, and updates were made to the rubric and to the questionnaire items until consensus was gained. In the second phase, seven letters were analyzed by the four investigators independently, and inter-rater reliability (IRR) was computed. IRR was assessed using a two-way random effects, consistency, single rating intraclass correlation coefficient (ICC). The resulting ICC was 0.69 (95% confidence interval 0.46–0.92) indicating fair to excellent agreement. Instruction item had the lowest reliability, and we updated the rubric and the scoring instructions. The final full analysis was conducted by one investigator (LD) with close supervision from another author (DS), including discussions when scoring was unclear, especially on item 6 instructions, which showed the lowest ICC for a single item.

In addition to the six items evaluating the letters’ quality, descriptive data was extracted regarding letter attributes and patients’ data. For letter attributes we counted the number of words and sentences in the narrative component. For patient data we extracted department, admission diagnosis, number of medical problems and number of medications, the last two reflecting the medical complexity of patients’ conditions.

The analysis was blinded, the investigators received coded and anonymized letters which gave no identification of student or patient identity or date. Letters were coded in random order so that first and last letters could not be identified.

Reliability of the assessment items was checked by Cronbach’s Alpha for internal consistency. The questionnaire reliability was high, 0.797.

### Data analysis

As the questionnaire items scores were not normally distributed, we used Wilcoxon Signed Ranks Test for comparison of paired data and Mann Whitney for unpaired. We did not use a correction to adjust probability values since this was an exploratory study employing simple statistic tests, where the result of each individual test is of importance [35].

The final overall score for each letter was the mean of the three category scores: telling the narrative, plain and clear language and structure. We checked for correlation of complexity of patients’ medical condition driven from patients’ data, and letter attributes with the final score, to find if they mediate the overall score.

## Results

Forty letters were submitted from the first rotation and 44 letters from the last, 84 letters overall. We paired letters where they were written by the same student groups. As a few students had switched groups through the year, ten letters could not be paired, giving a total sample size of 37 pairs, 74 letters written by 87 students. Thirty-three letters (45%) were from surgical admissions, 17 (23%) from obstetrics and gynecology 19 (26%) from pediatrics and a further 5 (7%) from orthopedic and internal medicine. The ten letters that were excluded as they could not be paired scored no differently from the letters included in the analysis for patients’ data, number of words and number of sentences.

When the paired letters were compared, the later letters scored higher than the first letters for all items. Significant increase in score was found for three items: telling the narrative Z = -2.853, *p* = 0.004, language and style Z = -2.732, *p* = 0.006, and medication table structure Z = -2.180, *p* = 0.029. The overall score significantly increased between the first and last letter, from 3.53 ± 0.8 to 4.08 ± 0.6, Z = -3.43, *p* = 0.001 (Table 2).

**Table 2:** Comparison of first and last letters for: Plain language writing quality, patients’ data, and letters attributes

Item	1st letter		3rd letter		*P*
	Mean (SD)	Median	Mean (SD)	Median	
Assessment Items					
1. Telling the hospitalization narrative	3.2 (1.1)	3.0	4.0 (1.2)	4.0	*P* = 0.004
2. Adequacy and clarity of medical terms	3.2 (1.2)	3.0	3.6 (1.2)	4.0	NS
3. Language and style	3.9 (1.2)	4.0	4.5 (0.8)	5.0	*P* = 0.006
4. Structure of the letter: medical problems	3.6 (1.2)	4.0	4.0 (1.0)	4.0	NS
5. Structure of the letter: Medication table	3.3 (1.3)	4.0	4.1 (1.1)	4.0	*P* = 0.029
6. Structure of the letter: instructions	4.2 (0.9)	4.0	4.4 (0.7)	5.0	NS
**Overall score**^**a**^	**3.5 (0.8)**	**3.5**	**4.1 (0.6)**	**4.2**	***P*** **= 0.001**
Patient Data					
No. medications listed	3.10 (2.96)	2.00	3.83 (3.02)	3.00	NS
No. of background diseases	2.62 (3.14)	1.00	2.54 (2.44)	2.00	NS
Letter attributes					
No. Sentences narrative	11.2 (7.8)	9.00	14.2 (10.3)	10.00	*P* = 0.042
No. words narrative	153.7 (108.0)	106.00	189.3 (116.3)	155.00	*P* = 0.027

^a^mean score of three factors: telling the narrative (item 1), plain and clear language (items 2,3 averaged) and structure (item 4–6 averaged)

We found no significant differences in the patient data, i.e. number of medications and background problems, indicating that latter patients’ conditions were as complex as earlier patients. The number of sentences and words in the narrative were significantly greater for later letters, indicating that students had written fuller explanations (Table 2).

In scoring the first letters, telling the narrative received the lowest score, followed by the plain and clear language category and the structure category. For the third letter, telling the narrative score improved so that it was equivalent to two other factors.

No relation was found between the overall score and number of words or sentences in the narrative, nor between the overall score and the complexity of the patients’ medical conditions, as indicated by number of problems or medications (Table 3), indicating they did not affect the final score.

**Table 3:** Pearson correlation coefficient between questionnaire overall score and letter attributes and patients’ data for 74 discharge letters

	No. Sentences narrative	No. words narrative	No. medications listed	No. of medical problems	Overall score
No. Sentences narrative		0.89**	0.04	−0.13	0.08
No. words narrative			0.11	−0.14	0.06
No. medications listed				0.52**	−0.08
No. of medical problems					0
Overall score					

** *p* < 0.01

## Discussion

Students’ written communication skills, as measured by our assessment tool, improved during the ETGAR course, from the first plain language discharge letter to the third. Our assessment tool was found to be reliable. Its score reflected the rubric and was not correlated to the complexity of patients’ medical conditions or to the number of sentences or words in the narrative which may affect the score of students’ reports and reflective writing [36].

Medical education programs in general lack appropriate training for writing discharge letters or referrals [37], and the few programs we identified were for residents [24], community based rotations [14] or volunteer programs [15]. The training program described in this paper may serve as a successful model for closing this gap. Incorporating communication skills into this patient care course, the clinical year students do not learn these skills as ‘stand-alone’, but within the context of real life. By meeting patients in hospital and at home, students had the opportunity to witness the impact of health literacy on patients’ health, its effect on health inequities and the role of clear communication in tackling them [26, 27].

Our program follows Kripalani and Weiss [34] recommendations for teaching health literacy education, including meeting patients and receiving feedback for the communication they practice, and contains the two main milestones for safe transition in care identified by Meade et al. [23] i.e. patient-centeredness and use of succinct written communication.

‘Telling the narrative’ received the lowest score for the first letters. Writing a good narrative involves firstly understanding the patient’s medical condition and treatment, and then transforming this complex picture to meet patients’ needs, and in a well-organized easy to understand and remember narrative. The quality of the original hospital discharge letters, which often miss relevant components or are written in an unorganized manner [37, 38] may be one of the barriers for students trying to accomplish this task. Establishing correct use of plain language is probably the first step of learning how to write medical information in plain language, to be followed by the more difficult task of setting the information as a narrative incorporating patient education within.

Our findings are similar to those of Bittner et al. [15], where an experience based training program including writing plain language reports for real patients improved the quality of the medical reports [15]. Our study took the work of Bittner et al. a stage further as it involved writing complex personal information rather than imaging reports, and assessed real-life written information given to patients and not an artificial pretest-posttest exercise.

Our study may have achieved more than a mere educational exercise. Improving patients’ understanding by providing them with quality discharge letters in plain language can positively affect patients’ health, since health literacy is associated with self-care behaviors [39], adherence to medication regimes [40] and mortality [9]. Reducing adverse events and improving self-care of poorly health literate patients has the potential to contribute to reducing inequity [18, 24]. Furthermore, the opportunity to discuss the plain language discharge letter with patients in their homes during a home visit exposed the students to some of the benefits of good communication around discharge.

The study does have some limitations. The entire student cohort was involved, as it was a required course, so we had no control group and therefore cannot assume causality. Since the students had no other written communication exercise, it is not likely the improvement may have accrued without the intervention, although increased clinical exposure may be a factor. A readability test would have augmented our results but unfortunately no such test is available in Hebrew. We found improvement in students’ plain language writing according to our guidelines and assessment tool, but further research is required to ascertain whether this improved patients’ understanding of discharge information. A larger number of letters from other cohorts are also needed to confirm our assessment tool validity and generalizability.

## Conclusion

In this study students improved their written communication skills following preparation of plain language discharge letters for patients whom they visited following discharge. This extended experience-based course, with repeated practice followed by Faculty feedback may have promoted learning. The assessment tool developed for this study was found to be reliable and was able to discriminate the discharge letter attributes.

The course and its accompanying assessment alongside the findings described in this paper may help direct other institutions to providing written communication training to undergraduate students in addition to verbal communication training.

## Data Availability

The datasets used and/or analysed during the current study are available from the corresponding author on reasonable request.

## Footnotes

1 ETGAR – the Hebrew word for challenge, is an acronym for health literacy, support and a bridge between medicine and community.
